# Implementing Individual Placement and Support (IPS) program of supported employment in Finland: Experiences on integrating the program in psychiatric care

**DOI:** 10.1192/j.eurpsy.2023.695

**Published:** 2023-07-19

**Authors:** K. Appelqvist-Schmidlechner, N. Sipilä

**Affiliations:** Equality, Finnish Institute for Health and Welfare, Helsinki, Finland

## Abstract

**Introduction:**

Mental health problems increase the risk for long-term sick leaves, early transitions to disability pensions and unstable career paths. Individual Placement and Support (IPS) is an evidence-based program integrated into psychiatric care aiming to support individuals with serious mental illness in finding employment. One key element in the implementation of the program is that the program is adequately integrated into the psychiatric care. However, knowledge on implementation challenges as well as best practices experienced by the practitioners, especially from the perspective of professionals in psychiatric care, is limited.

**Objectives:**

The Finnish Individual Placement and Support Evaluation Study (2020-2023) aims at investigating the implementation, feasibility as well as perceived benefits and outcomes of IPS program. The present study focuses on experiences on implementation of the program in the psychiatric care.

**Methods:**

Both quantitative and qualitative data from different stakeholders have been and will be collected. The data collection will be finished at the end of 2022. For the present study, data among professionals (psychiatric nurses and psychiatrists) in the psychiatric care has been and will be collected using questionnaires (n=58) and individual interviews (n=17). Among IPS employment specialist delivering the program, the data have been / will be collected using focus group interviews (6 focus groups, n=29) and workshops.

**Results:**

The preliminary findings of the study show that the key elements in successful implementation of the IPS program into the psychiatric care are sufficient information about the program among professionals in the psychiatric care, adequate flow of information between IPS employment specialists and psychiatric care (including regular meetings) and facilities promoting the co-operation (including physical space and common information system). Majority of professionals in psychiatric care reported that the client-related communication with the IPS employment specialist had been active (81%) and adequate (76%).

**Conclusions:**

Successful implementation and integration of the IPS program into psychiatric care requires seamless cooperation and communication between mental health professionals and IPS employment specialists, especially in cases when the IPS employment specialists do not have access to patient information due to confidentiality legislation.

**Disclosure of Interest:**

None Declared

